# A systematic review of the long-term outcome of early onset schizophrenia

**DOI:** 10.1186/1471-244X-12-150

**Published:** 2012-09-19

**Authors:** Lars Clemmensen, Ditte Lammers Vernal, Hans-Christoph Steinhausen

**Affiliations:** 1 Research Unit at Glostrup Center of Child and Adolescent Psychiatry, Ndr. Ringvej 69, 2600, Glostrup, Denmark; 2 Present address: Research Unit for Child and Adolescent Psychiatry, Aalborg Psychiatric Hospital, Aarhus University Hospital, Moelleparkvej 10, Aalborg, DK, 9000, Denmark; 3 Clinical Psychology and Epidemiology, Institute of Psychology, University of Basel, Missionsstrasse 60/62, Basel, CH, 4055, Switzerland; 4 Department of Child and Adolescent Psychiatry, University of Zurich, Neptunstrasse 60, Zürich, H-8032, Switzerland

**Keywords:** Early onset schizophrenia, Childhood onset schizophrenia, Long-term course, Outcome, Prognosis

## Abstract

**Background:**

The current review analyzes the long-term outcome and prognosis of early onset schizophrenia based on previously published studies in 1980.

**Methods:**

A systematic search of articles published in the English-language literature after 1980 identified a total of 21 studies, which included 716 patients who were either suffering from early onset schizophrenia (EOS) or both EOS and other psychotic disorders (MIX). The authors of the current review scored the outcome as either “good,” “moderate,” or “poor.” The mean age of onset in these studies was <18 years.

**Results:**

In general, the outcome in studies with EOS is worse than the outcome in MIX studies. Only 15.4% of the patients in EOS studies versus 19.6% of the patients in MIX studies experienced a “good” outcome. In contrast, 24.5% of the patients in EOS studies versus 33.6% in MIX studies experienced a “moderate” outcome, and 60.1% in EOS studies versus 46.8% in MIX studies experienced a “poor” outcome. The authors identified various significant effects on outcome. In EOS, the findings were significantly affected by sample attrition, indicating that in studies with a high dropout rate, fewer patients experienced a “moderate” outcome, and more patients experienced a “poor” outcome; however, the effect sizes were small. Furthermore, the effects were also small and more favourable for specific functioning measures, as opposed to more global measures, small to moderate in terms of worse outcomes for follow-up periods >10 years, small to moderate for more unfavourable outcomes in males, and small to large for worse outcomes in studies including patients diagnosed before 1970.

**Conclusions:**

In contrast to the adult manifestation, the early manifestation of schizophrenia in childhood and adolescence still carries a particularly poor prognosis. According to these aggregated data analyses, longer follow-up periods, male sex, and patients having been diagnosed before 1970 contribute predominantly to the rather poor course of EOS.

## Background

Traditionally, schizophrenia has been perceived as a disorder with high rates of chronicity and deterioration over time. Some recent studies have shown better prognosis of the disorder [1,2]. Typically, the onset is in early adulthood with less frequent manifestation in adolescence and rare onset in childhood. In the literature, the definition of early onset schizophrenia (EOS), or adolescent onset schizophrenia, varies with studies defining it as onset before age 17–21 [1,3-18].

Quite similarly, the age of onset in very early onset schizophrenia (VEOS), or childhood onset schizophrenia (COS) also varies across studies with definitions before 12–15 years of age [3,9,15,17,19-25]. The most common definition of EOS is onset before age 18, and the most common definition of VEOS is onset before age 13.

While adult onset schizophrenia (AOS) has been studied in great detail for many decades, research on EOS and VEOS is still more limited, partly due to its low prevalence and the fact that EOS was not recognized in the diagnostic systems before the introduction of DSM-III. The prevalence of schizophrenia in children and adolescents is rather low, with estimates of VEOS varying between 1 in 10.000 [21], 1 in 30.000 in children before age 13 [13], and 1.4 in 10.000 before age 15 [26]. Among patients with schizophrenia, a Finnish study found that 4.7% had onset at or before age 18 [27].

The nosological status of schizophrenia in children has been discussed for many years. In the DSM-II, the category of childhood schizophrenia referred to both psychotic and autistic disorders; however, the eminent studies by Kolvin et al. [28] made clear that schizophrenia in children had to be differentiated from autistic disorders. Since the appearance of the DSM-III, children with schizophrenia have been diagnosed with the same criteria as adults [23,29-31]. Both the stability and reliability of the diagnosis of EOS [31-35] as well as the validity of the diagnosis in children and adolescents are firmly established [1,36-39].

In 2005, more than 800 studies focused on the outcome of schizophrenia, irrespective of age at onset [40]; however, the majority looked at adult onset. Most studies on outcome of EOS have been restricted to small samples and/or short follow-up periods. The results are inconclusive across studies with some showing a prognosis resembling that of AOS but most reporting poorer prognosis [21,23,34,38,41]; only a few studies do not concur with this trend [42-44].

One of the more recent cohorts was studied at the Early Psychosis Prevention and Intervention Centre (EPPIC) in Melbourne, Australia [45]. This cohort contained patients with mixed early and adult onset psychosis. At a rather short mean follow-up of 18 months, the study found no significant difference between early and adult onset on outcome variables related to remission. In a more recent analysis, the follow-up period in the EOS subsample was extended to a mean of 6.9 years; the authors claimed that there was a better outcome in the EOS group compared to the adult onset group [42].

It has been suggested that differences in outcome across studies may be more to the degree of disability than in the rate of recovery [30]. Generally, there is agreement that the course of schizophrenia is rather heterogeneous among both adults and children [41,46,47]. Various predictors of outcome have been studied with no clear picture emerging due to a lack of replication studies. However, there is evidence that the diagnosis of VEOS predicts lower educational achievement, less independence both economically and emotionally, lower rates of employment, poor social relationships, and a continuing need of psychiatric care [21].

The current systematic review is focused on the analysis of the entire existing literature on the long-term outcome of EOS published since 1980 in English-language journals. We have chosen not to include studies published before 1980 because, regardless of their scientific validity at the time, they focused primarily on symptoms, they did not report on functional outcome in a standardized way, and they did not express shortcomings in terms of the studies’ participants. In addition to detailed descriptions, the current analysis is based on inferential statistical tests of aggregated data across studies in order to study both effects and prognostic factors. The report was written in accordance with the guidelines of the PRISMA statement [48].

## Methods

### Identification of studies

**Figure 1:**
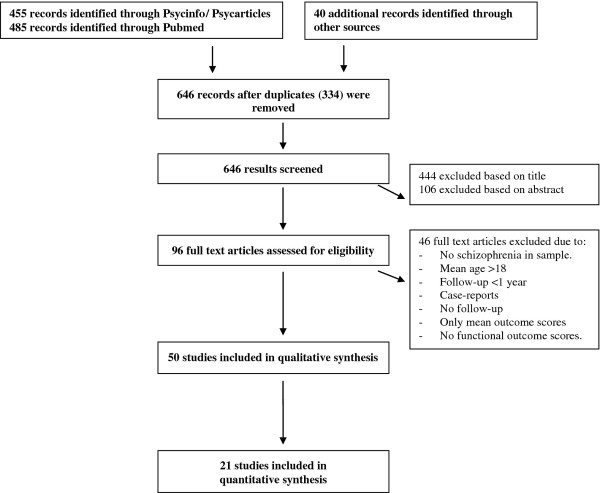
Flowchart of the literature search.

The literature search was carried out using the following databases: PsycINFO, Pubmed, and PSYCarticles. A search in Psycinfo and PSYCarticles for English-language articles published since 1980 using the criteria “AB = adolescent onset schizophrenia,” OR “childhood onset schizophrenia,” OR “very early onset schizophrenia,” OR “early onset schizophrenia” yielded 455 results. A search of publication titles and abstracts in PubMed based on the same terms and limitations yielded a total of 485 articles; 96 articles were chosen for further inspection. In addition, studies mentioned in previous review articles were also considered. The process of the literature search is shown in Figure 1.

Due to the interest in performing quantitative analyses based on inferential statistical tests, the following exclusion criteria were used: single case studies, studies reporting only on single or specific parameters (e.g., IQ or mortality) but no overall broad outcome measures allowing a classification into “good,” “moderate,” or “poor” outcome (see below), studies only reporting on mean outcome parameters, studies not based on internationally accepted diagnostic criteria (as reflected in the ICD and the DSM), studies with follow-up time <1 year, and studies with poor description of outcome criteria (e.g., no global functioning scores). In the case of duplicate publishing, data from the sample were included only once in the data set with the study that included the latest selected assessment. The analysis included both retrospective and prospective studies.

A mean age of ≤18 years was required. The majority of the studies only included patients aged <18 years with just a few studies also including 18 year olds [5,11,49,50], one study including patients aged 19 years [6], and one study [32] including a few patients aged 20 years at the time of onset; however the latter study was included because of a mean age at onset of 16.8 years. Studies reporting data on pure EOS and studies reporting combined data on EOS and other psychotic illnesses (MIX) were included in the analyses.

A total number of 21 studies were suitable for analysis [1,5,6,8,11,15,18,23,29,32,38-41,49-55].

### Outcome measures and effect variables

All data was collected from published material only. The studies were categorized as reporting outcome by use of either a General Functioning Scale (GFS, including Global Assessment of Functioning (GAF, [56])), Children`s Global Assessment Scale (CGAS, [57]), and Global Assessment Scale (GAS, [58]) or Study-Specific Functioning (SSF) outcomes. All GFS studies used scales running from 0 to 100. A total of 10 studies used GFS scales [5,23,32,39,40,50-53,55]. The GFS studies were categorized as a “poor” outcome (score ≤ 50), “moderate” outcome (score 51–70), or “good” outcome (score >70). Out of these ten studies, five had deviating definitions of the three outcome categories. As described in Table 1, four studies used other cut-offs for “poor,” “moderate,” and “good” [5,23,40,52]. One study [51] even divided the more generally used class of “poor” outcome into deteriorated (< 30) and minimal improvement (30–50). These two groups were combined into “poor” outcome (< 50), whereas all other deviating ratings were taken directly into the analysis.

**Table 1:** Overview of the 21 Studies

						**Sex**					**Outcome (%)**		
**Authors**	**Diagnosis**	**Period of Diagnosis**	**N**	**Dropout N (%)**	**Age at onset (yrs.)**	**Female**	**Male**	**Duration of follow-up (yrs.)**	**Outcome criteria**	**Original outcome ratings**	**Good**	**Moderate**	**Poor**
						**N (%)**	**N (%)**						
Hassan et.al (2011)	SZ, psychosis NOS	2003-2010	37	14 (27)	Mean = 12.2	23 (62)	14 (38)	Mean =3.2	CGAS: Good: ≥ 70		27.0	48.7	24.3
									Moderate 40-70				
									Poor: ≤ 40 and partial or no remission.				
Ledda et al. (2009)	SZ	1992-2002	15	2 (12)	Mean = 15.1	9 (53)*	8 (47)*	5	GAF*		11.8	60.0	27.6
Reichert et al. (2008)	SZ & SZ-AFF	1990-2000	27	59 (80)	Mean = 15.5	8 (30)	19 (70)	Mean = 13.4	Employment	3,7% university study	22.2	51.8	25.9
										18,6% regular work			
										48,1 sheltered work			
										25,9% unable to work			
										3,7% unemployed			
Remschmidt et al. (2007)	SZ	1920-1961	38	0 (0)	5-14	23 (61)	15 (39)	Mean = 42	GAS:		5.8	23.7	60.5
									Good >71				
									Moderate: 41-70				
									Poor: <40				
Fleischhaker et al. (2005)	SZ	1983-1988	81	20 (20)	11-18	36 (44)	45 (56)	4-11	GAF:		19.80	38.20	42.00
									Poor: <40				
									Moderate: 41-70				
									Good >71				
Helgeland et al. (2005)	SZ	1963-1978	9	N.A	13-17	1 (11)	8 (89)	Mean = 28,1	Social disability (medication, means of income, living situation)	All on antipsychotic medication at follow-up, all on disablement benefits, none living in an ordinary home	0,00	0.0	100.00
Röpcke et al. (2005)	SZ, SZ-AFF, schizo-phreni-form disorder	1979-1988	39	16 (29)	Mean = 16	19 (49)	20 (51)	10.2-21.2	GAS:		21.00	28.00	51.00
									Good >60				
									Moderate: 51-60				
									Poor: <51				
Jarbin (2003a)	SZ	1982-1993	30	58 (66)	11.8 - 18.7	11 (37)	19 (63)	5.1-18.2	GAF (or employment if GAF not available)	79% very poor	3.00	0.00	97.00
										18% poor			
										3% good			
Hollis, (2000b)	SZ	1973-1991	51	17 (25)	Mean = 14.0	22 (43)	29 (57)	4-22	Remission at follow-up		12.00	40.00	48.00
Lay et al. (2000)	SZ & SZ-AFF (ICD-9)	1976-1987	65	31 (32)	11,5-17,9	38 (59)	27 (41)	10	Social disability (DAS-scale and global evaluation on a 6-points scale)	12,5% no dysfunction,	20.00	44.00	36.00
										7,8% minimum,			
										14,1% obvious,			
										29,7% serious,			
										31,3% very serious,			
										4,7% maximum dysfunction			
McClellan et al. (1999)	SZ		11	7 (39)	11-16	3 (27)	8 (73)	2	Course of illness and description of impairment		0.0	9.00	91.00
Aarkrog (1999)	SZ, SZ-AFF	1968-1976	28	N.A.	12-20 (M = 16.8)	7 (25)	21 (75)	17-26	GAS		3.6	17.9	78.5
Eggers et al. (1997)	SZ (DSM-III-R)	1925-1961	44	27 (38)	6-14	25 (57)	19 (43)	Mean = 42	Social disability (Eggers social scale)	1-2: Good remission GAS >70	25.00	25.00	50.00
										3-4: Moderate remission < GAS 51			
										5-6: Poor remission - < GAS 40			
Maziade et al. (1996)	SZ (DSM-III-R)	1968-1990	40	37 (48)	10-17	13 (33)	28 (67)	14.8	GAS		5.00	15.00	80.00
Werry et al. (1994)	SZ, Schizo-phreni-form disorder	1968-1990	53	41 (36)	7-17	22 (42)	31 (58)	4.3	Living situation		20.7	17.00	62.30
Rund 1994	SZ (ICD-9)	1980-1990	24	0 (0)	13,1-17,9 (Mean = 16)	8 (33)	16 (67)	2	GAS		0	21.0	79.0
Cawthron et al., 1994	SZ (ICD-9)	1975-1986	9	10 (53)	14-18	-	-	2-13	Adult Personality Functioning Assessment	Seven (78%) continuously ill. None of these employed or married; extremely poor social functioning. The two recovered patients (22%) were ill for only 2% of the follow-up period.	22.00	0.00	78.00
Asarnow et al. (1994)	SZ	1980-?	18	3 (14)	6-11,3	5 (24)	13 (76)	2-7	CGAS	28% good outcome CGAS >60, 28%	28.00	28.00	44.00
									>60 = good				
									51-60 = moderate				
										moderate improvement CGAS 51–60, 28%			
									<51 = poor				
										minimal improvement CGAS <51, 17% deteriorating CGAS <41			
Gillberg et al. (1993)	SZ (DSM-III / ICD 9)	Born 1960–1982.	23	0 (0)	13-19	9 (39)	14 (61)	11-17	Overall register data outcome	13% overall possibly good	13.00	9.00	78.00
										9% intermediate outcome			
										78% extremely poor			
Krausz et al. (1993)	SZ, mood disorders, psychoses (PSE)	1972-1978	55	6 (10)	14-18	28 (51)	27 (49)	11-16	Mental and social handicaps rated according to Brown (1966),	20% inpatient, 26%	29.6	18.5	51.9
										seriously handicapped			
										16% handicapped but employed			
										26% not handicapped			
										12% no findings			
Inoue et al. (1986)	EOS and acute psychotic episode (DSM-III)	1971-1981	19	N.A.	10-17	9 (47)	10 (53)	3	Ability to work	47% unable to work	16.00	37.00	47.00
										16% limited work ability			
										21% working at a lower level than previously, 16% working as before			

If not otherwise specified: GAF: Global Assessment of Functioning >70 = Good; 70-51 = Moderate; <51 = poor. SZ: Schizophrenia. SZ-AFF: Schizoaffective disorder. CGAS: Children Global Assesment of Functioning Scale. GAS: Global Assessment Scale. N.A. = Not assessed. PSE = Present State Examination. *Based on N at baseline.

The SSF outcomes were also rated as “poor,” “moderate,” or “good” depending on the outcome as defined in these studies and shown in Table 1. In two studies [6,29] ratings were based on outcome scales, whereas the ratings in the remaining nine studies [1,8,11,15,18,38,41,49,54] were based on categorical outcome measures. Based on the above-mentioned three categories of outcome and using the same cut-off scores, the three authors of the current study performed the ratings independently in each study. There was full consensus among the three authors in the independent ratings of 19 studies, and after two authors agreed in the remaining two studies, full consensus was also reached for these remaining two ratings.

Dropout rates were comprehensively described in 17 studies and ranged from a minimum of 0% [6,23,55] to a maximum of 59% [15]. Reasons for dropout included untraceable subjects, subjects refusing to participate, death, moving out of the area, and suicide. One study, however, included suicide as a measure of outcome [50], but since most studies did not do so, suicides were subtracted from the data in this particular case in order to have consistent criteria for all ratings.

A total of five predicting variables were considered in the analyses as to their effect on the outcome measures: drop-out rate, type of measures of functioning, duration of follow-up, sex, and time period when patients had been diagnosed. Duration of follow-up was grouped into 1–10 and >10 years. The cut-off was chosen to obtain comparable sample sizes. If the duration of follow-up did not fit into one of these two outcome groups due to varying length of follow-up within the sample, the mean duration was used for classifying the study [5,38,49,51].

In one study, there was no information on sex distribution [49], and only a minority of studies reported outcomes stratified for sex [1,11,41,52]. Multiple studies noted sex differences without reporting stratified data. Time period of diagnosis considered studies including patients diagnosed before and after 1970 (<1970+) [1,6,18,23,29,32,49] and studies with all patients diagnosed in 1970 and later (≥1970) [5,8,11,15,34,36,38,40,41,52,53,55].

Finally, diagnoses were considered by dividing the data-set into studies containing only patients with EOS and studies including both patients with schizophrenia and patients with other psychotic disorders, i.e., psychosis (MIX).

### Statistical analyses

The three categories of “good”, “moderate”, and “poor” were calculated in percentages and rounded to the nearest decimal. In order to take into account the large variation in sample sizes, weighted percentages were calculated by weighting each reported rate with the size of the study group. All analyses were based on adjusted sample sizes at follow-up assessments rather than actual sample sizes after patient recruitment.

Due to consistent and significant deviation of the data from the normal distribution, non-parametric tests were used in the analyses. The effects of the four predicting variables mentioned above on the three outcome measures were analyzed using the Mann Whitney test with Bonferroni adjustments of p-values correcting for multiple testing. Considering five tests, findings were significant at the p = 0.01 level and highly significant at p = 0.002. In addition, effect sizes were calculated using the formula of rho = z/√N), where 0.1 is indicating a small effect, 0.3 a moderate effect, and 0.5 a large effect. Data analyses were performed by use of the SPSS 20 (SPSS, Chicago).

## Results

### Study characteristics

The current review is based on 21 studies containing 716 patients at follow-up. Detailed information on study characteristics and outcome findings is provided in Table 1. The sample sizes ranged from 9 to 81 patients with a mean group size of 44.4 (SD = 19.4). There were considerable differences in design, group size, methods, duration of follow-up, type of evaluation, and missing data. Diagnostic classification changed considerably over the period in which the studies were conducted given the fact that patients had been diagnosed over a wide time period ranging from 1920 to 2010. Since the 1990s, there has been an increasing reliance on DSM-IV and ICD-10 criteria. In 16 studies consisting of 592 patients, the mean age at onset was 14.9 (SD = 1.6) years; five studies reported only age ranges [6,8,11,49,51].

The mean duration of follow-up varied between 1.5 and 42.0 years (mean = 14.4; SD = 11.4). In 20 studies based on 707 patients, a total of 394 males (56.5%) were included. Repeated follow-up assessments were based on six samples and findings were described in nine articles [8,11,29,35,41,53,54,59,60]. Unfortunately, the data from these studies are not suited for repeated measurement analysis because both the sample sizes between follow-up periods (except [11]) and the duration of follow-up differed considerably. The total group of studies (N = 21) was divided into a group of EOS studies (N = 422) and a group of MIX studies (N = 294).

In addition to the various descriptive parameters, Table 1 contains columns reporting the outcome criteria used in the various studies, the original outcome ratings, and the outcome (in%) divided into the three categories of “good,” “moderate,” and “poor,” as calculated and rated by the us, which we based on the data in the preceding column containing the original outcome ratings.

### Outcome in samples of pure EOS vs. mixed psychotic disorders

As shown in Table 2, studies only containing EOS patients came up with a rate of 15.4% with a “good” outcome, whereas 24.5% experienced a “moderate” outcome, and 60.1% experienced a “poor” outcome. In the MIX samples, the figures were 19.6% with “good” outcome, whereas 33.6% experienced a “moderate” outcome, and 46.8% experienced a “poor” outcome. In each outcome category, though, the variation across studies proved to be remarkably high.

**Table 2:** Outcome by diagnoses based on 21 studies (N = 716)

**Percentages of subjects by diagnosis**														
**Outcome variable**	**EOS**				**Mixed**						**Analysis**			
	**Mean**	**SD**	**Range**	**Median**	**Mean Rank**	**Mean**	**SD**	**Range**	**Median**	**Mean Rank**	**U**	**z**	**p**	**rho**
	**N = 422**				**N = 294**									
Good	15.4	7.7	0-28	15.8	300.05	19.6	9.1	0-29	21.0	442.40	37368	−9.08	<.001	0.34
Moderate	24.5	14.6	0-60	23.7	299.75	33.6	12.9	18-52	37.0	442.83	37241	−9.13	<.001	0.34
Poor	60.1	18.9	27-100	60.5	410.59	46.8	17.8	24-79	47.0	283.73	40051	−8.09	<.001	0.30

There were significant differences in outcome between the EOS and the MIX samples. A significantly greater proportion of the MIX samples experienced a “good” or “moderate” outcome compared to the pure EOS samples. Consequently, the percentage of patients with a poor outcome was smaller in the MIX samples than in the EOS samples. All effect sizes were moderate.

### Effects of drop-out rates in the samples

Dropout rates in 17 studies ranged between a minimum of 0% and a maximum of 59%. This distribution was dichotomized at the median, and studies were classified as having a high (>28%) or a low (<28%) dropout rate. The effect of the attrition on the three outcome parameters was assessed by Mann Whitney tests and showed highly significant differences in the “moderate” and “poor” outcome groups but not in the “good” outcome groups (see Table 3). The rate of “moderate” outcomes was significantly higher in the low attrition samples compared to the high attrition samples, whereas the opposite was the case in the “poor” outcome group with a higher rate of poor outcomes in the high attrition samples; however, the effect sizes were small. In contrast, the three studies with a dropout rate of 0% all experienced high numbers of “poor” outcome [6,23,55], ranging from 60.5% to 79%.

**Table 3:** Outcome by attrition rate based on 18 studies (N = 660)

	**Percentages of subjects by dropout rate**											
**Outcome variable**	**Low dropout (<28%)**				**High dropout (>28%)**				**Analysis**			
	**Mean**	**SD**	**Median**	**Mean Rank**	**Mean**	**SD**	**Median**	**Mean Rank**	**U**	**z**	**p**	**rho**
	**N = 342**				**N = 318**							
Good	18.8	8.2	19.8	324.72	17.0	8.0	20.7	340.77	52400.000	−1.081	n.s.	0.04
Moderate	32.0	12.8	38.2	370.67	25.2	15.6	25.0	291.96	42007.500	−5.301	<.001	0.21
Poor	49.1	15.3	48.0	293.44	57.7	21.3	51.0	373.99	41703.500	−5.423	<.001	0.21

### Effects of the measures of functioning

In order to assess the effect of measures of functioning, studies based on GFS were compared to those using SSF measures. As shown in Table 4, there were highly significant differences in the outcomes based on these two measures of functioning in the “good” and “poor” outcome groups of the EOS samples and the MIX samples. In the latter sample, the outcome also differed significantly for the “moderate” outcome group. In the EOS samples, there were lower rates of “good” and “poor” outcomes in studies based on GFS compared to SSF outcomes. This was also true for the “moderate” outcome groups of the MIX samples. The effect sizes were small for all comparisons. In the 5 studies reporting a mean GFS in EOS patients at follow up based on a total of 199 patients [5,39,50,51,53], the grand mean weighted for sample sizes of these studies was 47.0.

**Table 4:** Outcome by measures of functioning based on 21 studies (N = 716)

**Outcome variable**	**Percentages of subjects by measures of functioning**											
	**GFS**				**SSF**				**Analysis**			
	**Mean**	**SD**	**Median**	**Mean Rank**	**Mean**	**SD**	**Median**	**Mean Rank**	**U**	**z**	**p**	**rho**
**EOS**	**N = 222**				**N = 200**							
Good	14.3	7.8	15.8	185.75	16.6	7.5	20.7	240.07	16486	−4.60	<.001	0.22
Moderate	27.0	12.8	28.0	220.02	21.7	12.8	17.0	202.05	20309	−1.52	n.s.	0.07
Poor	58.7	15.3	44.0	186.00	61.7	15.3	62.3	239.80	16540	−4.56	<.001	0.22
**MIX**	**N = 128**				**N = 166**							
Good	15.0	11.1	21.0	122.18	23.1	0.4	20.0	167.02	7383	−4.54	<.001	0.22
Moderate	30.5	12.1	28.0	131.75	36.0	1.0	44.0	159.64	8606	−2.82	.005	0.14
Poor	54.5	22.6	51.0	165.75	40.9	0.7	30.0	133.42	8287	−3.27	.001	0.16

### Effects of duration of follow-up

Findings that deal with the effect of duration of follow-up are presented in Table 5. In the EOS samples, the effect was highly significant for all three outcome groups. Moreover, there was a moderate effect size indicating that follow-up longer than 10 years was associated with a smaller proportion of patients with a “good” and “moderate” outcome and a larger proportion of patients with a “poor” outcome. In the MIX samples, differences for “good” and “moderate” outcomes were highly significant, and differences were significant for poor outcomes; however, the effect sizes were small. In these samples, the rate of both “good” and “poor” outcomes was increasing with longer follow-up periods, whereas the rate of “moderate” outcome was declining.

**Table 5:** Outcome by duration of follow-up based on 21 studies (N = 716)

**Outcome variable**	**Percentages of subjects by duration of follow-up**											
	**<10 yrs**				**>10 yrs**				**Analysis**			
	**Mean**	**SD**	**Median**	**Mean Rank**	**Mean**	**SD**	**Median**	**Mean Rank**	**U**	**z**	**p**	**rho**
**EOS**	**N = 187**				**N = 235**							
Good	19.2	5.9	19.8	269.28	12.4	7.6	12.0	165.52	11167.5	−8.74	<.001	0.43
Moderate	29.4	14.8	38.2	243.08	20.6	13.3	23.7	186.37	16067.0	−4.78	<.001	0.23
Poor	51.4	15.8	42.0	150.18	67.0	18.4	60.5	260.30	10505.5	−9.28	<.001	0.45
**MIX**	**N = 80**				**N = 214**							
Good	16.4	11.6	16.0	120.69	20.8	7.7	21.0	157.51	6415.0	−3.35	.001	0.20
Moderate	37.5	11.7	37.0	180.86	32.1	13.0	28.0	135.03	5891.0	−4.16	<.001	0.24
Poor	46.1	23.5	47.0	126.55	47.1	15.3	51.0	155.33	6884.0	−2.61	.009	0.15

### Sex effects

Direct calculations could only be made on the basis of five studies reporting separate results for males and females (4 with MIX and 1 with EOS patients). As Table 6 shows, highly significant differences were found for “good,” “moderate,” and “poor” outcome. These results indicate a generally less favourable outcome for males who less frequently than females experienced a “good” or “moderate” outcome and more frequently experienced a “poor” outcome. The effect sizes were small to moderate.

**Table 6:** Outcome by sex based on 5 studies (N = 190)

**Outcome variable**					**Percentages of subjects by sex**							
	**Males**				**Females**				**Analysis**			
	**Mean**	**SD**	**Median**	**Mean Rank**	**Mean**	**SD**	**Median**	**Mean Rank**	**U**	**z**	**p**	**rho**
	**N = 92**				**N = 98**							
Good	17.6	10.6	24.0	82.41	23.2	11.7	30.4	107.79	3304.0	−3.22	<.001	0.23
Moderate	23.2	19.3	25.0	68.80	37.3	37.3	46.0	120.56	2052.0	−6.56	<.001	0.48
Poor	59.2	23.2	74.0	107.78	39.5	39.5	36.0	83.97	3378.	−3.03	.002	0.22

To further investigate the effect of sex in a larger sample, we compared 6 studies with <50% males to 14 studies with >50% males; findings are shown in Table 7. Differences were significant to highly significant on the various levels of outcome. There is a clear indication that both EOS and MIX studies containing a majority of males generally experienced a less favourable outcome. The proportion of “good” and “moderate” outcomes was lower in studies based on a male predominance, whereas the proportion was higher in the “poor” outcome groups.

**Table 7:** Outcome by sex proportions based on 20 studies (N = 707)

** Outcome variable**	**Percentages of subjects**											
	**<50% males**				**>50% males**				**Analysis**			
	**Mean**	**SD**	**Median**	**Mean Rank**	**Mean**	**SD**	**Median**	**Mean Rank**	**U**	**z**	**p**	**rho**
**EOS**	**N = 97**				**N = 316**							
Good	19.4	5.3	15.8	262.34	13.9	7.9	13.0	190.01	9958	−5.26	<.001	0.26
Moderate	29.9	12.9	25.0	238.20	23.6	14.4	17.0	197.42	12300	−2.97	.003	0.15
Poor	50.7	11.0	50.0	175.80	62.5	19.9	62.3	216.58	12300	−2.96	.003	0.15
**MIX**	**N = 157**				**N = 137**							
Good	25.0	4.3	27.0	188.68	13.3	9.2	16.0	100.31	4290	−8.99	<.001	0.52
Moderate	36.1	13.1	44.0	160.27	30.7	12.1	28.0	132.86	8749	−2.79	.005	0.16
Poor	38.9	10.7	36.0	119.96	56.0	19.9	51.0	179.07	6340	−6.02	<.001	0.35

### Effects of time period of diagnosis

The data-set allowed a dichotomization into two groups of studies, namely, those including patients diagnosed before and after 1970 and those where all patients in the sample were diagnosed in 1970 or later. Table 8 provides a comparison of the outcome of these two groups. In the EOS samples, there is a highly significant decline of “good” outcomes in all patients diagnosed in or after 1970; however, the effect is only small. In contrast, there are large effects indicating that the proportion of “moderate” outcomes increased significantly, and the proportion of “poor” outcomes decreased significantly over time. Taking all three levels into account, the overall outcome improved significantly over time.

**Table 8:** Outcome by time period of diagnosis (N = 705)

**Outcome variable**	**Percentages of subjects by period of diagnosis**											
	**<1970+**				≥**1970**				**Analysis**			
	**Mean**	**SD**	**Median**	**Mean Rank**	**Mean**	**SD**	**Median**	**Mean Rank**	**U**	**z**	**p**	**rho**
**EOS**	**N = 216**				**N = 195**							
Good	16.2	7.6	15.8	228.03	15.8	7.1	19.8	181.60	16302.0	−3.987	<0.001	0.19
Moderate	17.2	7.3	17.0	137.25	24.9	15.8	38.2	282.15	6210.0	−12.452	<0.001	0.61
Poor	66.6	12.9	62.3	274.75	59.3	20.3	44.0	129.85	6210.0	−12.447	<0.001	0.61
**MIX**	**N = 28**				**N = 266**							
Good	3.6	0.0	3.6	38.50	19.6	7.9	21.0	158.97	672.0	−7.220	<0.001	0.42
Moderate	17.9	0.0	17.9	14.50	33.6	12.5	37.0	161.50	.0	−8.810	<0.001	0.51
Poor	78.5	0.0	78.5	256.50	46.8	15.3	47.0	133.03	672.0	−7.220	<0.001	0.42

**Note:** <1970+: studies including patients diagnosed before and after 1970.

≥1970: Studies containing patients diagnosed in 1970 and later.

There was only one MIX study containing patients diagnosed before 1970. On the other hand, there were clear moderate time period effects indicating highly significant improvements with increasing proportions of “good” and “moderate” outcomes and decreasing proportions of “poor” outcomes.

## Discussion

This is the first systematic review on the outcome of EOS that is covering all suitable studies published in the English-language literature since 1980. The analyses were based on statistical tests measuring both the general outcome and the effects of clearly defined predictors. The review focuses on general trends; one has to consider that the studies report rather diverse findings, though in part, this diversity may be explained by the pronounced heterogeneity of the schizophrenia syndrome itself [61,62]. Furthermore, the distributions of the main outcome variables of “good,” “moderate,” and “poor” differ depending on the measurements and definitions used in the various studies.

The main findings are the following: (a) the outcome for EOS is relatively poor and less favourable than in MIX samples; (b) samples with high dropout rates report less “moderate” and more “poor” outcomes, even though the effect sizes are small; (c) the effect sizes of measures of functioning are also small, which can be attributed to the fact that in EOS samples global measures of functioning are associated with less “good” and “poor” outcomes than specific measures of functioning; however, in the MIX samples, specific measures of functioning are associated with better outcomes on all three levels; (d) in EOS, the effect of duration of follow-up shows less favourable outcomes after more than 10 years of follow-up, whereas in MIX samples, the longer follow-up is associated with more “good,” less “moderate,” and more “poor” outcomes; (e) the outcome in both EOS and MIX samples is less favourable in males; and (f) the outcome is better in patients who had been diagnosed in more recent decades. In the subsequent paragraphs, these major findings will be put into perspective.

### General outcome

In the current review, we discovered that 15.4% of EOS patients experienced a “good” outcome, 24.5% experienced a “moderate” outcome, and 60.1% experienced a “poor” outcome. Clearly, these findings indicate that EOS is still a mental illness with a rather unfavourable prognosis; this conclusion is in accordance with previous reviews [5,7,12,21,31,63,64]. On the other hand, these previous reviews were based on non-aggregated data, and they did not employ rigorous data analyses as the authors do in the current review.

Furthermore, from the current analyses, it became evident that studies of patients with EOS show a worse prognosis than studies containing both patients with EOS and patients with other psychotic disorders (MIX). Unfortunately, separate analyses of the outcome of the various psychotic disorders were not feasible. In addition, differences in time points of measurement in the two samples may have been operant. Nevertheless, the different outcome in the two groups may serve as some indirect evidence that other psychotic disorders, i.e., schizoaffective disorders, schizophreniform or bipolar disorders with psychotic features, take a less serious course in terms of chronicity and functioning because all analyses based on the mixed psychotic samples showed a less severe outcome than the pure EOS samples. This finding is in accordance with similar studies in adults [2,65].

When considering the impact of dropout rates, the general findings on outcome may be only slightly different than one would expect without any attrition in the samples. In samples with high attrition rates, patients with a “moderate” course of the disorder were less likely to be followed up, and those with a “poor” outcome were more likely to show up at follow-up assessments at the various sites, whereas there was no attrition effect on the rate of “good” outcomes. In contrast, it is unclear whether the rate of “poor” outcomes would be different. On the one hand, our analyses showed that the rate of “poor” outcomes declined significantly with low attrition rates. On the other hand, three studies without any attrition showed an increased rate of “poor” outcomes. However, one has to keep in mind that the effect sizes for attrition were only small. High dropout rates are very common in psychiatric services with estimated rates ranging from 20 to 60% [66], which proves to be in line with the findings in the current review with dropout rates between 0 and 59% and a median of 29%.

### Impact of age at onset

In contrast to EOS, the outcome in studies of adult patients is generally more favourable [5]. Hegarty et al. [67] reviewed 320 adult studies from 1895 to 1992 (more than 50,000 patients in total) and found that approximately 40% improved considerably during follow-up. Jobe & Harrow [2] reviewed nine North American studies and the WHO-coordinated International Study of Schizophrenia (ISoS), all with a follow-up period of 10 years or longer, and concluded that, although adult patients with schizophrenia as a group have a worse outcome than other psychiatric patients, only a few patients show a progressive deteriorating course; depending on the strictness of the criteria used for diagnosis, 21-57% experience a “good” outcome. The ISoS compared long-term follow-up studies (10–15 years) from 14 culturally diversely treated incidence cohorts and four prevalence cohorts, totaling 1633 subjects, and found that approximately 50% experience a “good” outcome [65].

A recent international study that examined outcome after three years of follow-up in adult outpatient schizophrenia (N = 11.078 from 37 countries) found that 66% achieved clinical remission measured with the CGI, whereas only 25.4% achieved functional remission defined as good social functioning for 6 months in terms of occupational/vocational status, independent living and active social interactions [68]. There were large regional differences in the study. Patients in Europe were less likely to achieve clinical remission but were doing better in regards to functional remission. The general outcome both in the EOS group and the mixed psychotic group in the current review clearly shows that schizophrenia and psychosis originating in childhood and adolescence on average follows a worse course than AOS. In comparison to other disorders originating in childhood or adolescence, EOS stands out by way of its particularly poor course. For instance, the outcome seen with eating disorders is much better as is shown by similar types of analyses by the senior author [69,70].

This conclusion is also supported by a recent large cohort study from Israel with 12.071 participants. This study found that earlier onset corresponds linearly with the severity of the course of the disorder and appears to have some prognostic impact [71]. Young age at onset might have a detrimental effect on outcome because of impact at very crucial times of development and neurobiological maturation in childhood and adolescence, which prove to have more lasting effects in terms of both cognitive and psychosocial impairments [1,32,35,72].

So far, unfortunately, there are only a small number of outcome studies based on VEOS patients only, with an over-representation of females, whereas there are more studies with a varying range of age at onset within the defined EOS age range. Furthermore, there is not a single study based exclusively on patients with adolescent onset schizophrenia; thus, there are no real solid data for a comparison of the outcome of VEOS. Clearly, more detailed analyses will be needed. Given the low prevalence rates of VEOS, only collaborative studies across several sites could arrive at sample sizes needed for a differential look at the effects of age, sex, clinical features, or treatment effects on the outcome of VEOS.

### The impact of the measures of functioning

The current study is the first to make use of analyzing the impact of specific measures of functioning. Not surprisingly, the advent of global measures of functioning since the seventies also had an effect on the studies of the outcome of EOS. In contrast, a few studies continued with an older tradition to define study-specific functioning outcomes. Thus, a comparison of these two different traditions became possible. In the pure EOS studies, there were relatively small effect sizes, indicating that studies based on the more recent GFS arrived at slightly lower rates of both “good” and “poor” outcomes and no differentiation in the “moderate” outcomes than studies based on SSF outcomes. Accordingly, in EOS the overall pattern is clearly not more favourable for one of these two types of outcome. In the MIX studies, the effects were also small though more clearly showing a general pattern of less favourable outcomes based on GFS rather than on SSF assessments. Thus, the two analyses point to different findings in the two types of studies. In other words, the heterogeneity of the MIX samples favour the SFS outcomes in which the measurement might have tipped closer to the differences in the diagnostic composition of the samples.

However, this interpretation is only an assumption that needs further examination. Particularly, both the validity and the reliability of these measures need to be studied in greater detail. So far, this has been tested only in parts for some of the GFS measures in general child and adolescent psychiatry patients [73] but not specifically in patients suffering from schizophrenia. In particular, the GAF confounds symptoms and functioning with lower ratings driven by symptoms, so someone who is symptomatic but functional will receive a misleadingly low rating.

### The impact of intervention

In general, there is very little information on the impact of intervention on the outcome in EOS, even though all patient samples were seen clinically and received treatment. With the exception of a single study [72], all studies provided treatment as usual. In a recent intervention study with follow-up based on the Australian Early Psychosis Prevention and Intervention Centre (EPPIC) study, the authors found an increase in GAF score with a mean GAF score of 64 at follow-up [42]. By comparison, Oie et al. [74] found a mean GAS score of 47.7 in their EOS group containing 15 patients assessed when they were clinically stable on antipsychotic medication and followed up for 13 years. Moreover, Kao et al. [75] found a mean GAF score of 47 in 19 EOS patients after 1 year follow-up, and Gochman et al. [76] found a mean CGAS score of 43.6 after at least 8 years of follow-up. In the single intervention study included in the current review, the mean GAF score was 35 in the intervention group and 24 in the control group [55]. Only Ledda et al. [53] found a mean CGAS score of 62.1, which is quite comparable to the finding of the EPPIC study [42].

Nevertheless, in the latter study [42], the attrition rate was large (22/63) in the total EOS group and well explained only in a single person who committed suicide. It is unclear whether the 21 other patients that were not followed-up represent a subgroup with less favourable outcome because the authors did not provide a thorough attrition analysis. Thus, the claim of the authors that their outcome findings are superior to previous outcomes is not yet substantiated.

### The impact of duration of follow-up

The current analyses revealed, with small to moderate effect sizes, that across the three levels, the outcome deteriorated with longer follow-up periods (>10 years) in the EOS samples, but the association was rather curvilinear in the MIX samples with both “good” and “poor” outcomes increasing at the expense of “moderate” outcomes with longer follow-up periods. These differences again point to the already noted different course of the other psychoses, apart from schizophrenia. Nevertheless, the present findings need to be interpreted with caution because the two follow-up periods of ≤10 and >10 years are rather broad and reflect limitations of the data not allowing a more fine-grained analysis. Furthermore, the analysis was based on a series of cross-sectional rather than longitudinal studies. Unfortunately, among the 21 outcome studies of the present analysis, there are only six samples that were assessed repeatedly for follow-up and were described in 9 articles [8,11,29,35,41,53,54,59,60]. The study by Krausz and Müller-Thomsen [11] showed an increase in the proportion of “good” outcome from the first follow-up at 5 years to the second follow-up at 11 years (19 to 31%), whereas the rate of “poor” outcomes declined (74 to 59%) with a rather constant proportion of “moderate” outcomes (7 to 10%). These findings are in contrast to the findings of the current review. Lay et al. [41]) studied a mixed psychotic group that had been previously followed-up [35]. Unfortunately, approximately one third of the group had dropped out in between the two assessments, so it is unclear whether or not the slight shift from “moderate” to “poor” outcome (from 32 to 36%) is a valid finding. None of these longitudinal studies made use of inferential statistical tests of any significant change of the course of the disorder over time.

### The impact of sex

The current review supports the notion that male sex carries a less favourable prognosis in EOS but also in MIX samples. Nevertheless, as described in the methods section, there were profound limitations in the data for a proper analysis of sex effects. With a few exceptions [1,11,41,52,55], the vast majority of studies did not stratify outcome by sex; thus, two rather restricted types of analyses had to be performed. The direct comparisons of a small subsample suitable for direct comparisons clearly favoured females in terms of having a better outcome. The supplementary analysis based on a larger sample compared the outcomes of samples with either less or more than half of the samples being comprised of males. The findings were in line with the previous results indicating that male sex is a negative prognostic factor.

When looking at the various studies considered in the current review, one may see that some studies reported a tendency for worse outcome for males [1,5,6,8,11,15], but only two of these provided statistically significant differences [5,8]. One study found a specific “poor” outcome in females which proved to be not statistically significant [49]. In contrast, most of the 20 studies either reported no prognostic impact of sex [39,40] or did not specify or mention sex in relation to outcome measures [18,23,29,32,38,41,51,53,54]. One study noted that the risk of suicide was increased about 30 times in males [50].

In the 21 studies listing the distribution of sex, the average proportion of males was only 55%, a surprising discovery given that schizophrenia usually has an earlier onset among males than among females [17] and that late onset after age 45 is more common among females [24]. Especially with regard to VEOS onset, the literature points to a male predominance [12] with a ratio of approximately 2–2.5:1 [17,24,64]; however, in the two studies of VEOS, female sex was dominant in both series of patients [23,29]. In conclusion, there is some indication of a potential sex bias in the outcome studies in terms of containing more females than expected. Potential explanations include a higher dropout rate of males from outcome assessments due to less compliance and/or a higher mortality rate.

### The impact of time period of diagnosis

The time span of the original diagnosis of the patients varied enormously between 1920 and 2010. During this period, major changes in the understanding of schizophrenia including the nosological classification, assessment, and intervention took place. Thus, our analysis took potential time period effects into account. The data-set was dichotomized into studies containing patients diagnosed before or after 1970 and patients all diagnosed in 1970 or later. This grouping was not ideal because it was still based on considerable heterogeneity in terms of the time when the patients were diagnosed. Nevertheless, it represented a feasible and pragmatic approach and reflected the fact that some major changes in the classification of schizophrenia both in the ICD and the DSM took place in the seventies.

The findings indicated that the overall outcome in EOS and even more clearly in MIX samples improved over time; thus, one may argue that the progress in treatment and rehabilitation of schizophrenia might have had a beneficial effect for those who were born and diagnosed later. In summary, one may also conclude that the overall relatively poor long-term outcome of EOS is, in part, due to the inclusion of studies containing patients who had been diagnosed many decades ago.

### Limitations

First, we decided to include only studies published after 1980, assuming that these studies would reflect a rather common international frame of understanding of the nosology of schizophrenia and psychoses. Even with this restriction, though, there was a large time span over which patients had been diagnosed. Even more importantly, there may have been general problems with the recruitments of the samples. There might have been a bias both at the time of the first clinical presentation and of follow-up assessments. The less severely affected patients with a rapid remission of symptoms may not have been included at the beginning of the studies. Furthermore, it is not fully clear which effect no attrition might have had in particular on the rate of “poor” outcomes.

In the current review, we have used the categories of “good,” “moderate,” and “poor” outcome. These categories are commonly used in the outcome literature of various mental disorders. While the three authors of the current review showed excellent convergence in the outcome ratings of the various studies regarding this classification, one may argue that the cut-offs of these three outcome groups are debatable. Nevertheless, our cut-offs (>70 = good, 51–70 = moderate, and <50 = poor) imply some face validity because they are clearly demarcating major thresholds in functioning on GFS measures. Six GFS studies were based exactly on these definitions, whereas five studies used slightly different definitions. Two studies [40,51] used a lower cut-off of >60 rather than >70 for the definition of “good” outcomes, whereas three studies [5,23,52] requested a lower cut-off of <40 for “poor” outcomes. Thus, these differences imply a less strict definition of the outcome, so our findings might have been slightly better if we had accepted these definitions. Even among these five studies, though, there is no fully congruent set of definitions. Thus, our procedure was not only plausible in terms of the construction of the various GFS measures but also served as a good compromise considering the heterogeneity of definitions of “good,” “moderate,” and “poor” outcomes.

Some of the limitations in reviews of schizophrenia, as stated by Jobe & Harrow [2] and Castle and Morgan [77], are also relevant for the current analyses. The comparability of follow-up studies is compromised by differing criteria for diagnosis and outcome variables, sample selection (i.e., bias between inpatient and outpatient indexing), varying duration of follow-up, differences in the American and European tradition of diagnostic approaches, and prospective and retrospective designs leading to different preciseness of data acquisition. Furthermore, many studies have used different assessments for diagnosis and outcome. Various studies have lost patients due to suicide, which were counted as drop-outs; however, one could argue that suicide in terms of outcome should be listed in the “poor” outcome group, as suggested by Jarbin [50].

Furthermore, the lack of any clear data on mortality rates in EOS and VEOS is a shortcoming of outcome studies that should be addressed in future studies. Since EOS and VEOS are very rare, patients often come from a large geographic area to the specialist research units; thus, some patients travel far to be part of the study. This might be a bias in terms of only the most affected individuals will travel this far to be part of a study, which also indicates that the patients who have the best outcome might drop out.

Finally, no firm conclusions can be made thus far as to the effects of interventions, and it is unclear whether the large variation is due to different interventions, varying clinical manifestations, or an interaction of both. As with the study of other disorders, research on the effects of intervention on course and outcome is most neglected. Further studies are clearly needed.

## Conclusions

This exhaustive analysis of the available evidence on the outcome of EOS and VEOS points to the still rather poor prognosis of early manifestations of schizophrenia. The outcome of schizophrenia is worse than for other psychotic disorders, which applies to both adult and early onset schizophrenia. In both AOS and in EOS, though, there are many individual differences and so the course and outcome of schizophrenia is rather heterogeneous. Further insight into the long-term course of EOS might result from refinements in the design of future studies. Most particularly, the course of the individual patient will ultimately profit from a better understanding of the causes and refined treatment of this serious disorder.

Future studies on the long-term outcome of EOS might benefit from the following: (a) commonly used diagnostic criteria and standardized assessments; (b) detailed description of sample characteristics; (c) low attrition rates of the sample; (d) repeated and long-term follow-up assessments with standardized instruments covering clinical symptoms and functioning; (e) detailed information on type and duration of interventions including their effects on outcome; and (f) the use of large aggregated samples. These samples might be identified in national registers so that a potential sample bias caused by local hospital recruitment might be avoided.

## Abbreviations

EOS: Early onset schizophrenia; VEOS: Very early onset schizophrenia; AOS: Adult onset schizophrenia; MIX: Studies including both EOS and other psychotic disorders; GFS: General functioning scale; SSF: Study-specific functioning; GAF: Global assessment of functioning; CGAS: Children’s global assessment scale; GAS: Global assessment scale.

## Competing interests

The authors declare that they have no competing interests.

## Authors’ contributions

LC carried out all statistical analyses, DL was responsible for the literature search, HCS was responsible for the design of the study. All three authors performed the ratings of the outcomes and contributed equally to the writing of the manuscript. All authors read and approved the final manuscript.

## Authors’ information

Both LC and DL are psychologists and are currently working on dissertation projects dealing with various clinical aspects of early onset schizophrenia. Both are specializing in clinical child and adolescent psychology within child and adolescent psychiatry departments in Denmark. HCS is a research professor in child and adolescent psychiatry, a professor emeritus in child and adolescent psychiatry, and an honorary professor in clinical child and adolescent psychology.

## Sources of funding

None of the authors received any funding for this study.

## Pre-publication history

The pre-publication history for this paper can be accessed here:

http://www.biomedcentral.com/1471-244X/12/150/prepub
